# The Impact of Cyclodextrins on the Physiology of *Candida boidinii*: Exploring New Opportunities in the Cyclodextrin Application

**DOI:** 10.3390/molecules29153698

**Published:** 2024-08-05

**Authors:** Rita Márton, Márk Margl, Lilla Kinga Tóth, Éva Fenyvesi, Lajos Szente, Mónika Molnár

**Affiliations:** 1 Department of Applied Biotechnology and Food Science, Budapest University of Technology and Economics, 1111 Budapest, Hungary; martonr@edu.bme.hu (R.M.); marglmark97mm@gmail.com (M.M.); kingatth99@gmail.com (L.K.T.); 2 CycloLab Cyclodextrin R & D Laboratory Ltd., 1097 Budapest, Hungary; eva.fenyvesi@cyclolab.hu (É.F.); lajos.szente@cyclolab.hu (L.S.)

**Keywords:** bioactivity, biofilm formation, *Candida boidinii*, cyclodextrins, quorum sensing

## Abstract

Cyclodextrins, commonly used as excipients in antifungal formulations to improve the physicochemical properties and availability of the host molecules, have not been systematically studied for their effects and bioactivity without a complex active substance. This paper evaluates the effects of various cyclodextrins on the physiology of the test organism *Candida boidinii*. The research examines their impact on yeast growth, viability, biofilm formation and morphological changes. Native ACD, BCD, randomly methylated α- and β-CD and quaternary ammonium α-CD and β-CD were investigated in the 0.5–12.5 mM concentration range in both static and dynamic systems. The study revealed that certain cyclodextrins exhibited notable antifungal effects (up to ~69%) in dynamic systems; however, the biofilm formation was enhanced in static systems. The magnitude of these effects was influenced by several variables, including the size of the internal cavity, the concentration and structure of the cyclodextrins, and the contact time. Furthermore, the study found that CDs exhibited distinct effects in both static and dynamic systems, potentially related to their tendency to form aggregates. The findings suggest that cyclodextrins may have the potential to act as antifungal agents or growth promoters, depending on their structure and surrounding environments.

## 1. Introduction

Yeasts play a primary role in our lives. They are responsible for important ecosystem services and serve as bioprotective agents for health. These microorganisms are extensively utilized in diverse areas of biotechnology due to their versatile metabolic capabilities and well-understood genetic characteristics. Yeasts are also pivotal in biotechnologies related to the production of alcoholic beverages and play a crucial role in numerous food manufacturing processes, including those involved in coffee, chocolate, and table olive production) [1,2]. The presence of various yeast species, including pathogens, can pose potential health risks ranging from mild discomfort to severe illnesses [3]. *Candida* species are commonly studied from this perspective. The most prevalent infections include oral and vaginal candidiasis, but the greatest risk is posed to immunocompromised individuals [4,5]. The taxonomic diversity within the genus *Candida* has led to an expanding range of biotechnological applications for these yeasts. Various *Candida* species are employed as adjuvants in cheese production or as starters for coffee, cocoa, vegetable, meat, beer, and wine fermentations [6].

Despite being a less familiar member of the *Candida* species, *C. boidinii* has been isolated from various products of food processes such as wine fermentation and olive manufacturing, as well as natural environments including soil, seawater and sugar-rich tree species, etc. *C. boidinii* has also been identified as one of the most suitable yeasts for xylitol production [7]. Due to the broad taxonomic boundaries, the genus *Candida* has been the subject of research for numerous years; among these, studying quorum sensing (QS)—mainly with *C. albicans*—is particularly important. Quorum sensing is a cell-to-cell microbial communication mechanism in which microorganisms release signaling molecules into their environment, which are then detected by cells through their specific receptors [8,9]. The direct modulation of quorum sensing processes, including fungal communication, represents a novel approach to either impede or augment cell-to-cell communication, which is vital for microorganisms to adapt to their surroundings.

In recent years, cyclodextrins (CDs) have been shown to efficiently modulate bacterial communication in several Gram-negative bacteria [10,11,12,13,14]. Cyclodextrins (CDs) are capable of forming inclusion complexes due to their ring structure and dual polarity, which means they are suitable for reversibly binding certain guest molecules, improving their water solubility and biological accessibility, while the hydrophobic cavity acts as a binding site for hydrophobic molecules [15,16,17,18,19]. This CD-modulated quorum quenching (QQ) is a novel approach, and the available information on this topic refers mainly to their effect on processes regulated by bacterial communication. However, an unexplored but promising option is the use of cyclodextrins to interrupt or enhance cell-to-cell fungal communication; the comparative effect of various CD derivatives on the QS-regulated processes and pathogenesis in the *Candida* yeast system has not been assessed so far. CDs are often used as supporting compounds in antifungal formulations in order to change and improve the stability and solubility of the active ingredient. Nevertheless, some researchers propose that these cyclic oligosaccharides may also exert an influence on fungal physiological processes as bioactive compounds. One potential explanation for this is that cyclodextrins may form inclusion complexes with membrane lipids and cellular cholesterol [20,21,22,23]. Another explanation for the antifungal efficacy of cyclodextrins lies in their ability to mimic membrane-active compounds. Cyclodextrin derivatives, such as hexaamino-α-CD, heptaamino-β-CD, and octaamino-γ-CD, have demonstrated antifungal properties by mimicking the membrane-active characteristics of polymyxin B. The mechanism involves binding to anionic lipids on fungal membranes, disrupting membrane integrity, and forming channels, ultimately leading to the destruction of the cytoplasmic membrane [21,24]. Nevertheless, the available literature on the effects of cyclodextrins on yeasts applied alone without host molecules is limited and insufficient.

Currently, there are only a few examples that have not aimed to assess the effects of cyclodextrins alone on these fungal species. However, due to the importance of cyclodextrins as possible bioactive components and as potential regulators of fungal QS processes, we have conducted a complex systematic preliminary study on the time- and concentration-dependent effect of various CDs on *Candida boidinii* test organisms. Despite its high biotechnological potential, *C. boidinii* is one of the less-studied *Candida* species.

The main objective of our research was to explore the bioactive potential of native free CDs and their most widely used derivatives to attenuate or intensify the fungal growth and the quorum sensing mediated biofilm formation processes and non-QS mediated processes as well. One of our goals was to assess the impact of cyclodextrins on the growth and viability of *C. boidinii*. Additionally, we investigated their influence on the QS-mediated biofilm formation since previous studies demonstrated that cyclodextrins can complex the main fungal quorum-sensing molecules like farnesol and tyrosol [25,26,27]. One of the main hypotheses of our complex study was that certain CDs can influence the growth of *C. boidinii* and may also affect QS-regulated biofilm formation, presumably due to the complexation of the signal molecules. Based on our previous experience with the application of CDs, we anticipate that the interaction will be influenced by the unique structural characteristics of CDs.

## 2. Results

### 2.1. Effect of Cyclodextrins on C. boidinii in Dynamic Systems

**Figure 1:**
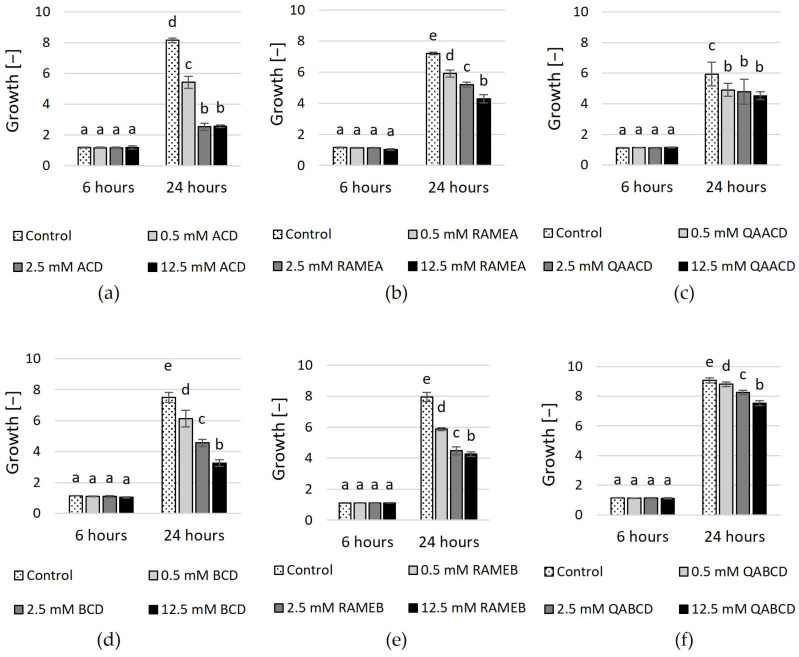
Effect of incremental (**a**) ACD, (**b**) RAMEA, (**c**) QAACD, (**d**) BCD, (**e**) RAMEB, and (**f**) QABCD concentrations on microbial growth in the dynamic system after 6 and 24 h of exposure time. Statistical significance (*p* < 0.05) is marked by lowercase letters, where a indicates the smallest value. Values signed with the same letter indicate that there was no significant difference between them. Data represent the averages of five replicates.

The effect of native ACD, BCD, randomly methylated α- and β-CD (RAMEA, RAMEB) and quaternary ammonium α-CD and β-CD (QAACD, QABCD) on the physiology of *C. boidinii* was tested within the 0.5–12.5 mM concentration range in both static and dynamic systems. The optical density measurements, which were used to determine cell growth within the population, were obtained via spectrophotometry following 6 and 24 h of incubation. The increasing concentration of the tested cyclodextrins impacted the growth, as illustrated in Figure 1. (Percentage inhibition values compared to control are summarized in Supplementary Table S1).

No significant effect was detected among the different treatments for any cyclodextrin after 6 h of incubation. However, after 24 h, concentration-dependent effects were observed regarding the inhibition of growth. The highest inhibition was exerted by ACD after 24 h (~69%). Inhibition was also noted in the case of RAMEA and QAACD, with values of ~40% and ~24% at the highest concentrations, respectively.

In the case of BCD and its tested derivatives, the highest inhibition (~57%) was achieved after 24 h with 12.5 mM BCD. The derivatives exhibited less growth inhibition compared to the native CD; however, the inhibition also increased with the CD concentration. 12.5 mM RAMEB exhibited ~46% inhibition, whereas this value was ~17% in the case of QABCD after 24 h.

**Figure 2:**
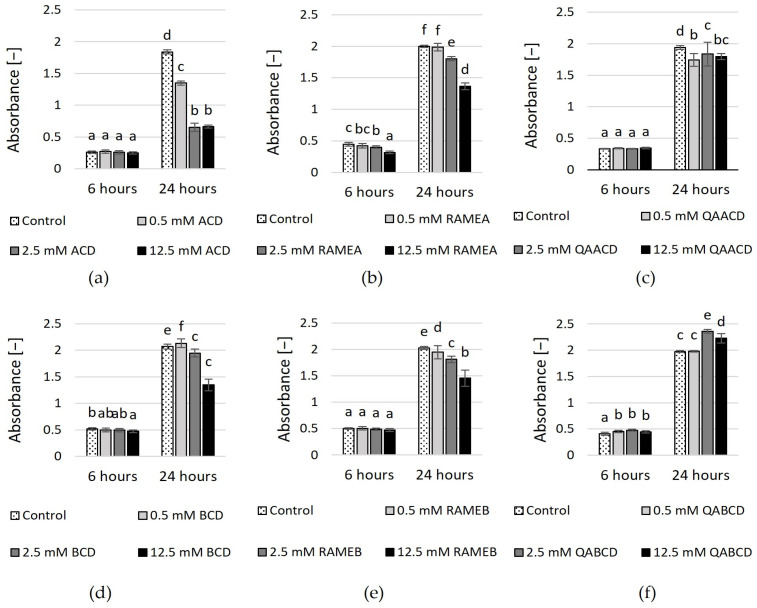
Effect of incremental (**a**) ACD, (**b**) RAMEA, (**c**) QAACD, (**d**) BCD, (**e**) RAMEB, and (**f**) QABCD concentrations on cell viability in the dynamic system after 6 and 24 h of exposure time. Statistical significance (*p* < 0.05) is marked by lowercase letters, where a indicates the smallest value. Values signed with the same letter indicate that there was no significant difference between them. Data represent the averages of five replicates.

The viability of the cells was measured by the tetrazolium reduction assay with MTT. Figure 2 shows the concentration-dependent inhibitory effects on cell viability.

Percentage inhibition values compared to control are summarized in Supplementary Table S2. The highest inhibition (65%) was exerted by 2.5 and 12.5 mM ACD after 24 h of exposure. The higher the dilution, the greater the cell viability in the presence of ACD, BCD, RAMEA, and RAMEB, while this tendency is not noticeable for trimethyl-aminopropyl derivates. QABCD exhibited a slight stimulating effect (<20%) on cell viability at concentrations of 2.5 mM and 12.5 mM after 24 h of contact time.

To investigate the effect of cyclodextrins on cell concentrations, the plate count method was also used, and the number of colonies formed (colony forming units—CFU) was determined. For the CFU data, similar to the growth determined by optical density, no significant effect was observed compared to the control during the 6-h incubation period However, after 24 h of incubation, the inhibitory effects were observed. In the case of ACD, all three treatments significantly inhibited growth. The highest concentration (12.5 mM) caused ~90% inhibition, while the 2.5 mM concentration inhibited growth by approximately 84% and the 0.5 mM concentration by approximately 49%. The effects of RAMEA were similar, significantly inhibiting CFU by 60–84%. For QAACD, the inhibition was less (approximately 30–53%), but the effects of the higher concentrations were still significant. In the case of BCD, both 2.5 mM and 12.5 mM concentrations significantly reduced the number of colony-forming units in a concentration-dependent manner, achieving reductions of approximately 62–82% compared to the control.

A similarly strong inhibitory effect was observed with RAMEB, where all concentrations significantly affected CFUs, with a concentration of 12.5 mM resulting in approximately 82% inhibition. In contrast, QABCD had no significant effect.

### 2.2. The Effect of Cyclodextrins on C. boidinii in Static Systems

**Figure 3:**
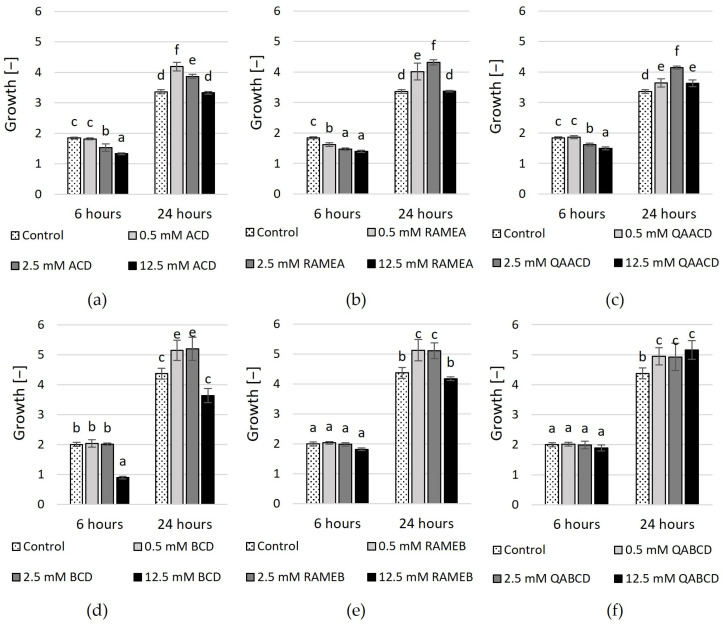
Effect of increasing concentrations of (**a**) ACD, (**b**) RAMEA, (**c**) QAACD, (**d**) BCD, (**e**) RAMEB, and (**f**) QABCD on microbial growth in the static system after 6 and 24 h of exposure time. Statistical significance (*p* < 0.05) is marked by lowercase letters, where a indicates the smallest value. Values signed with the same letter indicate that there was no significant difference between them. Data represent the averages of five replicates.

**Figure 4:**
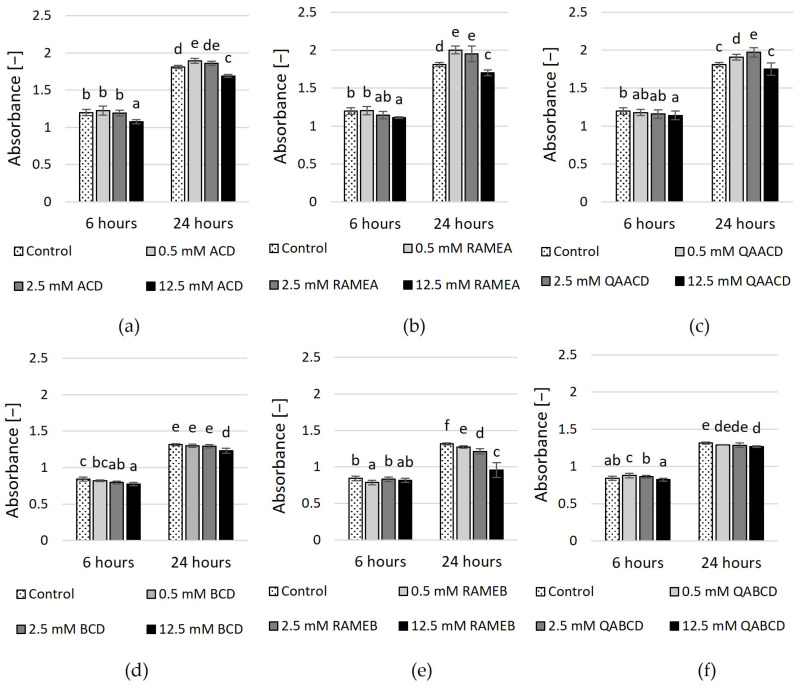
Effect of incremental (**a**) ACD, (**b**) RAMEA, (**c**) QAACD, (**d**) BCD, (**e**) RAMEB, and (**f**) QABCD concentrations on relative viability in the static system after 6 and 24 h of exposure time. Statistical significance (*p* < 0.05) is marked by lowercase letters, where a indicates the smallest value. Values signed with the same letter indicate that there was no significant difference between them. Data represent the averages of five replicates.

The outcomes of experiments conducted in the static system (Figure 3 and Figure 4, Supplementary Tables S1 and S2) exhibit variations when compared to the results obtained in the dynamic system. In contrast to the results obtained in the dynamic system, the inhibitory effect of cyclodextrins on growth varies in the static system. In the case of ACD and its derivatives (Figure 3), the results of RMANOVA analyses demonstrated the significant efficiency of ACDs. Both the contact time and the cyclodextrin treatments influenced the growth of the yeast. Furthermore, the effect of CD treatments was also significantly different over time. Besides, concentration-dependent but slightly inhibitory effects of ACDs on yeast growth were measured.

The highest inhibitory effect was obtained with 12.5 mM ACD, with 28% growth inhibition based on optical density. Slightly less inhibition in growth resulted from the application of RAMEA; a 24% reduction in growth was measured with 12.5 mM RAMEA. QAACD exhibited the least inhibitory effect, with a reduction of approximately 19% observed at 12.5 mM QAACD. However, after 24 h of incubation, all treatments (ACD, RAMEA, and QAACD) show stimulation compared to the control distilled water, which can reach up to ~29% in the case of RAMEA (2.5 mM).

Similar to ACDs, the BCD and its derivatives exhibited significant influences on the growth of *C. boidinii* based on the RMANOVA analyses. Both the contact time and the cyclodextrin treatments influenced the growth of the yeast. Furthermore, the effect of BCDs was also significantly different over time. However, with the exception of a few treatments, BCDs demonstrated stimulatory effects on growth after both 6 and 24 h.

The exception to this was 12.5 mM BCD, which resulted in ~55% inhibition after 6 h and nearly 17% inhibition after 24 h. In the other cases, mild growth stimulation effects (max. 18%) were observed.

The results of the MTT viability test (based on tetrazolium reduction measurement) are illustrated in Figure 4 and Supplementary Table S2. In the case of ACDs, only the highest concentrations of CDs exhibited a significant inhibitory effect after six hours. This was most pronounced for the 12.5 mM ACD, which exhibited an inhibitory effect of approximately 10%. Conversely, lower CD concentrations either caused a slight (11%) increase or had no significant effect. After 24 h, the results and tendencies were similar, with only the 12.5 mM ACD and RAMEA exhibiting a reducing effect in the MTT test. However, neither concentration reached the 10% inhibition threshold.

In addition, the viability test revealed that only the highest concentration of BCDs had a significant negative effect on yeast viability. After 6 and 24 h, 12.5 mM BCD was inhibitory, with a reduction of 8% and 6%, respectively. In comparison, 12.5 mM RAMEB showed an inhibitory effect of 27% at 24 h. These are only small reductions compared to the control, much smaller than what was observed with the dynamic system. However, the results of the RMANOVA analysis indicated that both the contact time and the cyclodextrin treatments significantly influenced the growth of the yeast.

Regarding the cell concentration (CFU) determined by the plate count method, the inhibitory effect of ACDs was less pronounced in a static system without shaking compared to the dynamic system (Supplementary Figures S1 and S2). Only the highest concentration of ACD and RAMEA resulted in inhibition, with ACD at around 61% and RAMEA at around 29%.

All other treatments stimulated growth, with stimulation reaching up to ~101%. In the case of BCD and QABCD, the treatments had no significant effect on CFUs. For RAMEB, the higher concentrations caused minimal stimulation.

**Figure 5:**
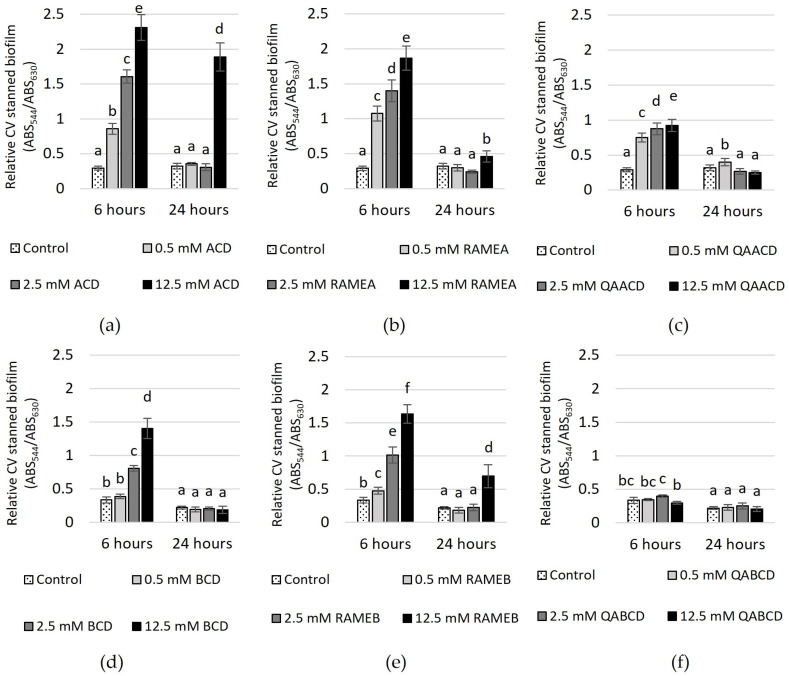
Effect of increasing (**a**) ACD, (**b**) RAMEA, (**c**) QAACD, (**d**) BCD, (**e**) RAMEB, and (**f**) QABCD concentrations on relative biofilm measured with crystal violet staining in the static system after 6 and 24 h of exposure time. Statistical significance (*p* < 0.05) is marked by lowercase letters, where a indicates the smallest value. Values signed with the same letter indicate that there was no significant difference between them. Data represent the averages of five replicates.

**Figure 6:**
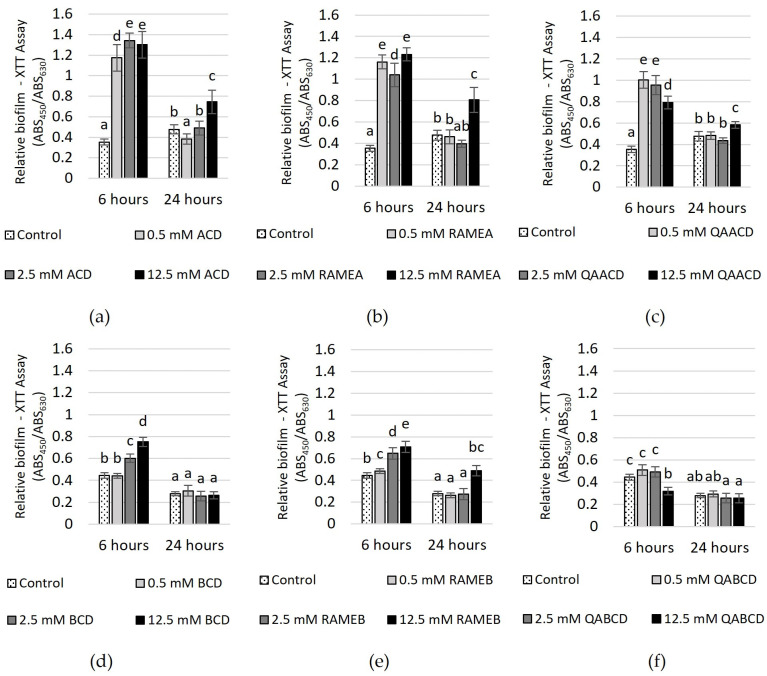
Effect of increasing (**a**) ACD, (**b**) RAMEA, (**c**) QAACD, (**d**) BCD, (**e**) RAMEB, and (**f**) QABCD concentrations of on relative biofilm measured with XTT assay in the static system after 6 and 24 h of exposure time. Statistical significance (*p* < 0.05) is marked by lowercase letters, where a indicates the smallest value. Values signed with the same letter indicate that there was no significant difference between them. Data represent the averages of five replicates.

In addition to evaluating the impact on growth and viability, we examined the effect of cyclodextrins on biofilm formation. We assessed the effects of the six cyclodextrins on *Candida boidinii* biofilm formation by measuring two endpoints. Quantitative determination of produced biofilm was performed using crystal violet staining, while measurement of metabolic activity of live cells within the biofilm was conducted using XTT staining. As illustrated by the figures (Figure 5 and Figure 6), the extent of biofilm stimulation was significantly influenced by the cyclodextrin concentration.

In the case of CV-stained biofilm (Figure 5), after 6 h of incubation, an increase in the CD concentration led to higher absorbance values, with the exception of QABCD, where no clear relationship was observed between the tested cyclodextrin concentration and the degree of biofilm formation. At a concentration of 12.5 mM, the highest stimulation of approximately 700% was observed in the case of ACD. Following this, RAMEA also showed significant stimulation at the same concentration, with an increase of approximately 540%. Subsequently, RAMEB, BCD and QAACD displayed stimulations of 390%, 320%, and 218%, respectively, all at 12.5 mM concentrations. After 24 h, the stimulation significantly decreased, with only 12.5 mM ACD maintaining approximately 490%.

In contrast, the stimulation did not exceed 220% after 24 h for the other treatments (12.5 mM RAMEB ~219%, 12.5 mM RAMEA ~43%, 0.5 mM QAACD ~25%), and in certain cases, there was no stimulation at all (BCD, QABCD).

The effect of increasing CD concentrations on the biofilm metabolic activity, as determined by the XTT assay, is illustrated in Figure 6.

After 6 h of incubation, significant increases in biofilm metabolic activity were observed with ACD, RAMEA, QAACD, and RAMEB treatments at concentrations ranging from 0.5 to 12.5 mM (232–279% with ACD, 194–248% with RAMEA, 124–184% with QAACD and 9–59% with RAMEB). In the case of BCD, only the two higher concentrations induced an increase, while QABCD did not stimulate activity. After 24 h, the stimulating effect diminished, similar to the observations in the CV assay. 

BCD and QABCD treatments showed no significant difference from the control. For ACD, RAMEA, QAACD, and RAMEB, stimulation was only induced by the 12.5 mM concentration, with the highest value reaching 75%.

**Figure 7:**
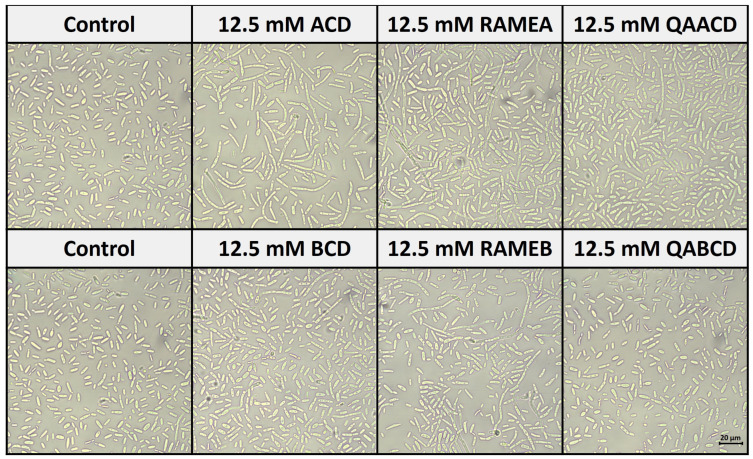
Effect of cyclodextrins on the morphology of *C. boidinii* after 24 h in the static system.

Figure 7 shows the effect of cyclodextrins on the morphology of *C. boidinii* after 24 h of incubation in the static system.

Based on our observations, all of the five CD treatments, except QABCD, increased the number of hyphal forms (formation of pseudohyphae) compared to the control distilled water. These results are consistent with the findings from the biofilm measurements. The most significant pseudohyphae formation was induced by ACD, which also resulted in the strongest biofilm formation.

## 3. Discussion

Cyclodextrins are widely used compounds with the ability to form inclusion complexes. They are often used to improve the effectiveness of antifungal drugs by enhancing solubility or increasing bioavailability. In our screening study, we took a different approach, aiming to evaluate the effects of cyclodextrins on fungal physiology in an independent manner. The main objective of our study was to systematically evaluate the time- and concentration-dependent effect of different CDs on the growth and viability of *C. boidinii* and to investigate their influence on QS-mediated biofilm formation. There are several approaches to studying the growth, viability and biofilm formation of microorganisms, which can be categorized according to different criteria.

In this preliminary phase of the research, we generally applied commonly used methods (e.g., optical density measurement, plate count technique, MTT-assay, crystal violet staining method for biofilm quantification) to characterize the yeast population’s growth and viability and quantify the biofilm formation. These methods have their own advantages and limitations and can be optimized for specific circumstances. However, as a primary method, these techniques can provide a reasonable basis for evaluating and characterizing the effect of cyclodextrins.

Two distinct experimental setups were employed to evaluate the impact of cyclodextrins on the growth and viability of *Candida boidinii*. In the dynamic system, we found that cyclodextrins within the 0.5–12.5 mM concentration range inhibited the growth of *C. boidinii.* The most significant inhibitory effect was observed in response to treatments with native ACD and BCD at a concentration of 12.5 mM, resulting in growth inhibitions of 69% and 57%, respectively. Although there is limited information on the cytotoxic and antimicrobial properties of cyclodextrins on yeasts, studies have been undertaken on other cell types.

Previous studies primarily concentrated on human cells [28,29,30,31]; however, these findings may not be directly applicable to yeasts due to variances in cellular structures and membranes. It has been demonstrated that cyclodextrins can interact with endogenous substances derived from the organism, tissue, or cells. These interactions primarily involve the formation of inclusion complexes with cholesterol and membrane lipids [28,29].

Kiss and colleagues [30] conducted an investigation into the cytotoxic and hemolytic properties of various CDs in relation to their cholesterol-solubilizing capacities, aiming to elucidate the mechanism of toxicity. The MTT cell viability test on Caco-2 cells revealed that the cytotoxicity of methylated BCDs was the highest, while certain ionic derivatives, such as QABCD, exhibited no toxicity even at a concentration of 200 mM. Their findings indicated that non-methylated cyclodextrin derivatives were less toxic than the methylated ones. Our own data also support this: QABCD had lower efficacy and lower toxicity (maximum 17%) compared to RAMEB (up to 46%). This trend was also seen in cyclodextrins containing six glucose units, as RAMEA induced approximately 40% growth inhibition at a concentration of 12.5 mM, whereas QAACD caused 24% inhibition under the same conditions. The differences observed in research conducted on mammalian cell lines are due to variations in the composition of the cell membrane. Fungi typically contain higher amounts of ergosterol in their cell membrane instead of cholesterol, leading to biological and chemical processes that can affect drug sensitivity. As many fungi and protozoa cannot survive without ergosterol, enzymes involved in its synthesis have become important targets in drug research [32].

The adverse effects observed in the dynamic experiment may also be due to the complexation and extraction of ergosterol, similar to the research of Teixeira and colleagues [22]. However, either changing the composition of the medium or complexing its constituents with cyclodextrin may also affect growth. The few studies in which the relationship between CDs and fungi is mentioned mainly highlight that these molecules improve the properties of antifungal agents [21]. In this respect, our research is completely unique.

The results obtained in the static system markedly differ from those observed in the dynamic system. This disparity in effects between the two systems may be attributed to the tendency of cyclodextrins to form aggregates. The aggregation of cyclodextrins, namely their self-assembly capability in water or other solvents, has been the subject of numerous studies. Typically, aggregation occurs through interactions of the hydrogen bonds of cyclodextrin molecules. The formation of aggregates is influenced by various factors, including cyclodextrin concentration, pH, temperature, and the presence of other solvents and additives [33,34]. The size and structure of the aggregates can vary, impacting their physical and chemical properties as well as their applicability. In our case, within the shaken system, the shear force likely led to a reduced tendency of cyclodextrins to form aggregates compared to the static system.

One of the aims of our research was to assess the time- and concentration-dependent effects of cyclodextrins on the biofilm formation capacity of *C. boidinii*. Given that biofilm formation in yeasts is a quorum-sensing (QS)-regulated process, influencing it has recently garnered attention due to the pathogenicity of certain species and their relevance in biotechnological applications. Comprehending the biofilm formation capability of *C. boidinii* and the influence of different molecules, such as cyclodextrins, on its behavior holds significant importance in biotechnological applications.

Based on our previous study [35], we applied a rapid, robust, inexpensive, high-throughput method (96-well microtiter plate model) with crystal violet staining for assessing and characterizing the effect of cyclodextrins on *C. boidinii* biofilm. This microtiter plate assay can be used to screen and pre-test the anti-biofilm effect of different substances in the different stages of biofilm formation without sophisticated equipment.

During the experiments, we observed that certain cyclodextrins induced a notable stimulation of biofilm formation, especially during the 6-h incubation period. Notably, native ACD elicited the most substantial stimulation, reaching nearly 700%.

Since the staining of the biofilm may not provide reliable information about the viability of the cells, only the extension and thickness of the matrix, additional complementary endpoint testing was also performed using the tetrazolium reduction assay with XTT to provide a more detailed picture of biofilm formation (e.g., biofilm metabolic activity). It has previously been shown that the XTT reduction assay shows a strong correlation between cell density and metabolic activity (cell viability), in contrast to crystal violet staining. In our study, the biofilm formation assay based on metabolic activity was more sensitive to differences in the extent of biofilm formation for most CDs than crystal violet staining, especially after 24 h of exposure.

Furthermore, our observations indicate that with the exception of QABCD, all other CD treatments resulted in an increase in the number of pseudohyphae forms compared to the control. These results are consistent with the findings from the biofilm measurements, with the most notable hypha formation observed in ACD. The morphological forms of *Candida* species, such as the transition between yeast, hyphae, and pseudohyphae, are primarily influenced by environmental conditions and biofilm formation. Key environmental factors include nutrient availability, pH levels, and temperature, with specific conditions such as acidic pH and body temperature promoting hyphal growth [36,37]. Biofilm formation further induces morphological changes, often favoring hyphal forms that enhance cell adhesion and biofilm stability. These adaptations enable *Candida* to thrive in varying environments and evade host immune responses [38].

The influence of cyclodextrins on yeast biofilm formation has received limited attention; nevertheless, investigations have been directed toward examining this phenomenon in other cell types, particularly in bacterial systems.

Berkl et al. [12] investigated the effect of native α- and β-cyclodextrins, along with their monomer derivatives, and epichlorohydrin-crosslinked polymers, on the biofilm formation and viability of *Pseudomonas aeruginosa*. The quorum-quenching ability of various CDs was investigated through QS-regulated biofilm formation. They found that cyclodextrins can influence bacterial communication by forming inclusion complexes with signaling molecules involved in bacterial communication, with the extent of this effect depending on the cyclodextrin’s cavity size and substituents.

The huge contrast between the inhibition of biofilm formation observed in their study and the stimulatory effect noted in our investigations arises from the differences between prokaryotic and eukaryotic test systems. One such distinction is that distinct quorum sensing (QS) signaling molecules have been identified in the two test organisms: while the QS system in *P. aeruginosa* primarily involves N-acyl-L-homoserine lactones, tyrosol has been identified as the QS molecule in *Candida boidinii* [35].

A key finding of our research is the effect of cyclodextrins on biofilm formation. The formation of biofilms may also be influenced by the complexation and accessibility of tyrosol or farnesol by cyclodextrins, which indirectly affects biofilm formation, as both molecules play an important role in biofilm formation [9].

In yeasts, especially in *Candida* species, farnesol was found to have a key role in the morphological transition, hindering hyphae production, while tyrosol has the opposite function, promoting the transition from spherical cells to the formation of germ tubes. Given that cyclodextrin is capable of forming complexes with both molecules [25,26,27], it is plausible to suggest that it may exert a considerable influence on the processes (e.g., biofilm formation) regulated by QS. The observed morphological alterations induced by the addition of CDs are also evident in their capacity to influence biofilm formation. Therefore, understanding the factors that drive the association and dissociation of the cyclodextrin-signal complex is important for unlocking the background mechanisms and planning effective cyclodextrin-based systems for applications that influence yeasts’ physiological processes.

Our plans also include developing a biofilm testing methodology because our observation indicates that the effects of cyclodextrins on *C. boidinii* are notably influenced by agitation methods.

## 4. Materials and Methods

A series of studies were carried out at concentrations ranging from 0.5 to 12.5 mM to examine the time- and concentration-dependent effects of natural α- and β-cyclodextrins (ACD and BCD), as well as their random-methylated (RAMEA and RAMEB) and trimethylaminopropyl (QAACD and QABCD) derivatives on the growth, viability, and biofilm formation of *Candida boidinii*. The tested cyclodextrins were fine chemicals provided by CycloLab Cyclodextrin R&D Laboratory Ltd. (Budapest, Hungary).

### 4.1. Yeast Strain and Culture Conditions

*Candida boidinii* strain ATCC 18810™ (DSM 70026) (Leibniz Institute DSMZ, Braunschweig, Germany) was used in the experiments. The yeast was maintained aerobically at 25 °C as an agar slant culture in the laboratory on yeast extract-peptone-dextrose (YPD) media solidified with 2% agar. The ingredients of the YPD medium were 10 g yeast extract; 20 g bacteriological peptone; 20 g glucose (and 20 g agar) per 1 L distilled water. For the tests, 16 h old (overnight) shaken cell culture was prepared by inoculating one loopful of yeast colony to 30 mL YPD growth media.

### 4.2. Tested Cyclodextrin Molecules

Table 1 shows the abbreviations with the degree of substitution given in parenthesis, the average molecular formula, the molecular weight, and the solubility in water at 25 °C of the tested CDs.

For the experiments, the cyclodextrins were suspended in distilled water and sterilized in an autoclave. A series of dilutions were made using the sterile CD stock solutions (or suspensions) in concentration ranges of 2.5–50 mM. A fourfold dilution was applied in the test systems, resulting in a final concentration range of 0.5–12.5 mM. Sterile distilled water was used as a negative control for each measurement.

### 4.3. Incubation Conditions

Two types of experimental set-up were implemented. A 96-well microtiter plate was used during static measurements, while measurements were conducted in 100 mL bottles for the larger-scale dynamic system. The static system may be relevant, especially for studying the effect of cyclodextrins on biofilm formation, which can be problematic in both healthcare and various industrial processes. In such cases, specific surfaces coated with specific (immobilized) cyclodextrins may prove an effective solution. The dynamic system can be used for simple modeling of mixed (agitated) bioreactor systems, e.g., cyclodextrin-aided bioconversions and fermentations.

#### 4.3.1. Static System

For the experiment conducted in the static microtiter plate, 50 μL of the sample (CD solution or distilled water) was pipetted into the wells, and then 150 μL of fungal suspension was added. The plates were incubated in the dark at 22 °C.

#### 4.3.2. Incubation Conditions (Dynamic System)

The elements of the dynamic experiment were assembled in 100 mL sterile Schott bottles with breathable caps. A threefold dilution series of cyclodextrins was prepared from a 50 mM stock solution in sterile test tubes using sterile distilled water. Amounts of 37 mL of YPD nutrient solution, 12.5 mL of CD solution (or distilled water for the control), and 0.5 mL of fungal suspension were pipetted in the bottles. During measurements, the flasks were incubated in the dark, under agitation at 150 rpm, at 22 °C. Samples were taken from them at the specified time points and transferred to microtiter plates, with a total volume of 200 μL in each well.

### 4.4. Population Growth—Optical Density Assay and Plate Count Technique

The optical density of the test medium was measured to determine the growth of the microbial population and evaluate if the CDs had any cytotoxic effect. Optical density was measured directly after assembling the plates and following the incubation periods with a DIALAB ELx800 ELISA Microplate Reader (Dialab GmbH, Wiener Neudorf, Austria) at a wavelength of 630 nm. To assess the changes in cell concentration of *C. albicans* yeasts, the plate count technique was used by plating serially diluted samples from dynamic and static systems on YPD agar plates. The number of the developed colonies (Colony Forming Units–CFU) was counted after 72 h of incubation, and the results were given in CFU/mL.

### 4.5. MTT Assay

Cell viability was determined by 3-(4,5-dimethylthiazol-2-yl)-2,5-diphenyltetrazolium bromide (MTT) colorimetric assay. After the incubation and the optical density measurement, 30 µL of 1 mg/mL MTT solution was added to each well. Then, the plate was incubated for 15 min in the dark at room temperature, and the absorbance was measured at 490 nm using the DIALAB ELISA EL800 Microplate Reader (Dialab GmbH, Austria).

### 4.6. Biofilm Formation Assay

Two assays were used to quantify the production of *Candida boidinii* biofilms: the XTT (tetrazolium salt reduction) test and the crystal violet (CV) assay. The CV assay was carried out as O’Toole described it, with a few modifications [39]. A 200 μL suspension was pipetted into selected 96-well sterile polystyrene microplate wells and incubated for 6 or 24 h at 22 °C. After incubation, the growth medium was decanted, and the biofilm was gently washed twice in a water-filled tub to remove nonadherent cells. A volume of 250 μL of 99% methanol was added into the wells for fixation. Following a 15-min incubation period, the methanol was removed, and the plates were dried under laminar flow. Then, the wells were filled with 250 μL of a 0.1% aqueous crystal violet solution, and plates were incubated for 15 min to stain the cells. The excess solution was removed, and the washing steps were repeated carefully to avoid damaging the biofilm. To solubilize the biofilm-bound CV, 250 μL of a 30% acetic acid solution was added to the wells. 250 μL of the extract was moved to a new 96-well plate after a 15-min exposure. The absorbance was measured at 544 nm with a Fluostar Optima BMG Labtech microplate reader. This method enables measuring cells attached to both the bottom and walls of the 96-well plates. 2,3-bis(2-methoxy-4-nitro-5-sulfophenyl)-5-[(phenylamino) carbonyl]-2H-tetrazolium hydroxide (XTT) assay was carried out with the same experimental set up as in the case of the CV biofilm formation test.

The XTT colorimetry assay was performed as previously described [35]. For the XTT/Menadione solution, XTT (Sigma-Aldrich Inc. Budapest, Hungary) was dissolved in saline at 1 mg/mL XTT, and Menadione (Sigma-Aldrich Inc. Budapest, Hungary) was dissolved in acetone to give a concentration of 0.4 mM. Both were filter sterilized through a 0.22-μm-pore-size filter, and the XTT solution was mixed with the menadione solution at a ratio of 5 to 1 by volume. Following the incubation, the cell suspensions were aspirated, and the wells were washed five times with 250 μL of (phosphate buffer saline) PBS to remove nonadherent cells. Then, 250 μL of PBS and 15 μL of XTT/Menadione solution were added to each of the wells. The microtiter plate was then incubated in the dark for 2 h at 37 °C. Thereafter, 100 μL of solution was transferred to a new 96-well plate. 

A colorimetric change in the XTT-reduction assay was measured in a microtiter plate reader (DIALAB ELx800 ELISA Microplate Reader, Dialab GmbH, Austria) at 450 nm.

### 4.7. Morphological Investigations

Morphological investigations based on microscopic examination were carried out after 24 h of incubation on samples from the static system to analyze the particular morphologies of *Candida boidinii* exposed to cyclodextrins. To assess whether cyclodextrins can affect the yeast-to-hyphae transition, morphological alterations were visualized with a Nikon Eclipse SI microscope equipped with a TrueChrome 4K Pro camera and Mosaic™ V2.4 imaging software under 400× magnification (Auro-Science Consulting Kft., Budapest, Hungary), and representative images were captured.

### 4.8. Statistical Analysis

The experiments were conducted in five replicates, and the standard deviation from the mean was calculated. Repeated measures analysis of variance (RM ANOVA) was performed with TIBCO Statistica™ 13.5 (TIBCO Software, Inc., Palo Alto, CA, USA) software to analyze the effect of the cyclodextrins on the *C. boidinii* yeast. We aimed to study the influence of cyclodextrin concentrations, exposure time and their interactions on the growth, viability, and biofilm formation of *C. boidinii*.

The Mauchley sphericity test was applied to verify the criteria. The Newman–Keuls post-hoc test was then applied to assess whether the treatments had a significant effect at the *p* < 0.05 significance level. In all figures, significant effects are marked by letters in alphabetical order. Columns sharing the same letter imply that there is no significant difference between them. 

## 5. Conclusions

In this study, we aimed to investigate the effects of cyclodextrins with varying numbers of glucose units and different substitutions on the growth, viability and QS-regulated biofilm formation of *Candida boidinii* yeast. The principal aim of our discovery research was to investigate whether the selected cyclodextrins with different structures have a potential antifungal or growth-promoting effect; both approaches could be important from a human health, environmental or biotechnological point of view.

The experiments revealed the cytotoxicity and influence on biofilm formation and hyphal growth, with the extent of effects influenced by factors such as CD size, concentration, structure, and contact time. However, further investigations are necessary to understand the interaction mechanism of cyclodextrins both in the complexation of QS signals and in damaging the fungal cells. Our results indicate that cyclodextrins have the potential to be applied as antifungal agents or growth promoters, depending on their structure. Since CDs may affect the biofilm formation regulated by both farnesol and tyrosol, this can result in different effects over time. Understanding how cyclodextrins influence biofilm formation can aid in developing new biotechnological applications.

## Figures and Tables

**Table 1:** Abbreviations (with the degree of substitution given in parenthesis), average molecular formulas, molecular weights and water solubility of tested cyclodextrins.

	Abbreviation	Average Molecular Formula	Molecular Weight [g/mol]	Water Solubility [g/L]
Native α-CD	ACD	C_36_H_60_O_30_	975	145
Native β-CD	BCD	C_42_H_70_O_35_	1135	18
Randomly methylated α-CD	RAMEA (11)	C_36_H_60-n_O_30_ · (CH_3_)_n_	1127	>500
Randomly methylated β-CD	RAMEB (12)	C_42_H_70-n_O_35_ · (CH_3_)_n_	1303	>500
Trimethyl-aminopropyl α-CD	QAACD (2.5–4)	C_36_H_60-n_O_30_ · (C_6_H_15_ONCl)_n_	1466	>500
Trimethyl-aminopropyl β-CD	QABCD (3–4)	C_42_H_70_-nO_35_ · (C_6_H_15_ONCl)	1665	>500

## Data Availability

The original contributions presented in the study are included in the article (and Supplementary Material), further inquiries can be directed to the corresponding author.

## Supplementary Materials

The following supporting information can be downloaded at: https://www.mdpi.com/article/10.3390/molecules29153698/s1. Supplementary Table S1. Effect of incremental concentrations of ACD, RAMEA, QAACD, BCD, RAMEB, and QABCD on microbial growth (optical density) after 6 and 24 h of exposure time. The data represents the averages of five replicates. Supplementary Table S2. Effect of incremental concentrations of ACD, RAMEA, QAACD, BCD, RAMEB, and QABCD on cell viability (MTT-assay) after 6 and 24 h of exposure time. The data represents the averages of five replicates. Supplementary Figure S1. The number of colonies formed by *Candida boidinii* (Colony Forming Units—CFU) after 24 h of exposure time with different concentrations of CDs in the dynamic system: (a) ACD, (b) RAMEA, (c) QAACD, (d) BCD, (e) RAMEB, (f) QABCD. The data represents the averages of three replicates. Supplementary Figure S2. The number of colonies formed by *Candida boidinii* (Colony Forming Units—CFU) after 24 h of exposure time with different concentrations of CDs in the static system: (a) ACD, (b) RAMEA, (c) QAACD, (d) BCD, (e) RAMEB, (f) QABCD. The data represents the averages of three replicates.
