# Supplementary material for: The Impact of Cyclodextrins on the Physiology of Candida boidinii: Exploring New Opportunities in the Cyclodextrin Application
Source: Molecules. 2024 Aug 5;29(15):3698. doi: 10.3390/molecules29153698 (PMC11313686; doi:10.3390/molecules29153698)
Supplement: Supplementary file 1 [file molecules-29-03698-s001.zip › molecules-3064260-supplementary.pdf]

molecules

MDPI

Supplementary

The Impact of Cyclodextrins on the Physiology of *Candida boidinii*: Exploring New Opportunities in the Cyclodextrin Application

Rita Márton [1], Márk Margl [1], Lilla Kinga Tóth [1], Éva Fenyvesi [2], Lajos Szente [2] and Mónika Molnár [1,*]

[1] Department of Applied Biotechnology and Food Science, Budapest University of Technology and Economics, 1111 Budapest, Hungary; martonr@edu.bme.hu (R.M.); marglmark97mm@gmail.com (M.M.); kingatth99@gmail.com (L.K.T.)
[2] CycloLab Cyclodextrin R & D Laboratory Ltd., 1097 Budapest, Hungary; eva.fenyvesi@cyclolab.hu (É.F.); lajos.szente@cyclolab.hu (L.S.)
* Correspondence: molnar.monika@vbk.bme.hu

Supplementary Table S1. Effect of incremental concentrations of ACD, RAMEA, QAACD, BCD, RAMEB, and QABCD on microbial growth (optical density) after 6 and 24 hours of exposure time. The data represents the averages of five replicates.

	**Inhibition of microbial growth [%]**					
	Dynamic system			Static system		
	**ACD**					
	0.5 mM	2.5 mM	12.5 mM	0.5 mM	2.5 mM	12.5 mM
6 hours	1.8 ± 3.3	1.6 ± 3.8	-1.2 ± 9.2	1.5 ± 1.5	16.8 ± 6.7	28.0 ± 1.5
24 hours	33.6 ± 4.7	69.1 ±0.2	68.6 ± 0.1	-24.6 ± 4.1	-14.9 ± 4.8	1.0 ± 1.3
	**RAMEA**					
	0.5 mM	2.5 mM	12.5 mM	0.5 mM	2.5 mM	12.5 mM
6 hours	2.7 ± 2.1	1.9 ± 3.3	10.3 ± 4.3	12.1 ± 13.1	20.1 ± 1.9	24.2 ± 2.2
24 hours	17.9 ± 3.2	27.9 ± 2.2	40.4 ± 3.7	-19.5 ± 8.3	-28.5 ± 2.5	-0.3 ± 0.6
	**QAACD**					
	0.5 mM	2.5 mM	12.5 mM	0.5 mM	2.5 mM	12.5 mM
6 hours	-1.8 ± 2.1	-0.9 ± 2.2	-2.8 ± 1.8	-1.2 ± 2.7	12.2 ± 2.7	18.9 ± 2.9
24 hours	17.3 ± 6.9	19.4 ± 13.7	23.7 ± 4.3	-8.4 ± 3.9	-23.3 ± 1.6	-8.0 ± 3.4
	**BCD**					
	0.5 mM	2.5 mM	12.5 mM	0.5 mM	2.5 mM	12.5 mM
6 hours	3.7 ± 2.8	3.1 ± 4.3	8.7 ± 3.7	-1.8 ± 6.2	-0.5 ± 1.7	55.1 ± 2.2
24 hours	18.3 ± 7.2	39.2 ± 2.9	56.5 ± 2.8	-17.7 ± 7.8	-19.0 ± 8.8	16.7 ± 5.4
	**RAMEB**					
	0.5 mM	2.5 mM	12.5 mM	0.5 mM	2.5 mM	12.5 mM
6 hours	-0.3 ± 1.6	-0.5 ± 1.9	-0.7 ± 2.1	-1.7 ± 2.4	0.9 ± 2.8	9.1 ± 2.0
24 hours	26.0 ± 1.2	43.6 ± 3.3	46.3 ± 1.9	-17.9 ± 8.2	-17.0 ± 6.1	4.5 ± 1.4
	**QABCD**					
	0.5 mM	2.5 mM	12.5 mM	0.5 mM	2.5 mM	12.5 mM
6 hours	0.5 ± 1.2	0.8 ± 1.9	1.6 ± 4.1	-0.9 ± 3.1	0.5 ± 6.3	5.3 ± 5.0

24 hours	2.9 ± 1.7	9.0 ± 1.5	16.9 ± 1.9	-13.0 ± 6.5	-12.6 ± 10.4	-17.9 ± 7.1

Supplementary Table S2. Effect of incremental concentrations of ACD, RAMEA, QAACD, BCD, RAMEB, and QABCD on cell viability (MTT-assay) after 6 and 24 hours of exposure time. The data represents the averages of five replicates.

	**Inhibition of cell viability [%]**					
	Dynamic system			Static system		
	**ACD**					
	0.5 mM	2.5 mM	12.5 mM	0.5 mM	2.5 mM	12.5 mM
6 hours	-3.3 ± 10.9	1.4 ± 10.3	4.2 ± 6.6	-2.2 ± 5.0	0.6 ± 3.4	10.3 ± 2.1
24 hours	26.7 ± 1.8	64.5 ± 3.5	63.9 ± 1.3	-4.6 ± 1.9	-2.8 ± 1.6	6.5 ± 1.1
	**RAMEA**					
	0.5 mM	2.5 mM	12.5 mM	0.5 mM	2.5 mM	12.5 mM
6 hours	5.5 ± 7.8	10.9 ± 4.5	29.7 ± 4.6	7.2 ± 1.0	4.4 ± 4.0	-1.1 ± 5.0
24 hours	0.6 ± 2.9	9.7 ± 1.7	31.6 ± 2.8	5.8 ± 2.1	-7.9 ± 5.7	-10.1 ± 3.0
	**QAACD**					
	0.5 mM	2.5 mM	12.5 mM	0.5 mM	2.5 mM	12.5 mM
6 hours	-2.1 ± 2.6	0 ± 1.7	-3.5 ± 3.1	4.9 ± 4.7	3.3 ± 4.7	2.4 ± 3.5
24 hours	10.2 ± 5.3	5.3 ±9.8	7.3 ± 2.5	3.2 ± 4.5	-8.9 ± 3.4	-5.0 ± 2.0
	**BCD**					
	0.5 mM	2.5 mM	12.5 mM	0.5 mM	2.5 mM	12.5 mM
6 hours	3.7 ± 6.7	4.5 ± 6.1	8.3 ± 5.1	2.6 ± 1.3	5.5 ± 2.5	8.0 ± 2.8
24 hours	-2.9 ± 3.9	6.0 ± 3.6	35.0 ± 5.2	1.1 ± 1.8	1.6 ± 1.7	6.2 ± 2.5
	**RAMEB**					
	0.5 mM	2.5 mM	12.5 mM	0.5 mM	2.5 mM	12.5 mM
6 hours	0.3 ± 6.3	3.3 ± 4.1	6.2 ± 5.9	6.6 ± 3.9	1.3 ± 3.6	3.1 ± 3.6
24 hours	3.9 ± 6.1	10.6 ± 3.1	28.2 ± 7.6	3.2 ± 1.4	7.9 ± 2.8	27.2 ± 7.6
	**QABCD**					
	0.5 mM	2.5 mM	12.5 mM	0.5 mM	2.5 mM	12.5 mM
6 hours	-11.5 ± 4.8	-16.8 ± 4.8	-9.2 ± 6.0	-4.3 ± 3.6	-2.4 ± 2.1	2.2 ± 2.5
24 hours	-0.2 ± 0.8	-19.4 ± 1.9	-12.8 ± 4.6	1.8 ± 2.4	2.3 ± 2.4	3.5 ± 0.7

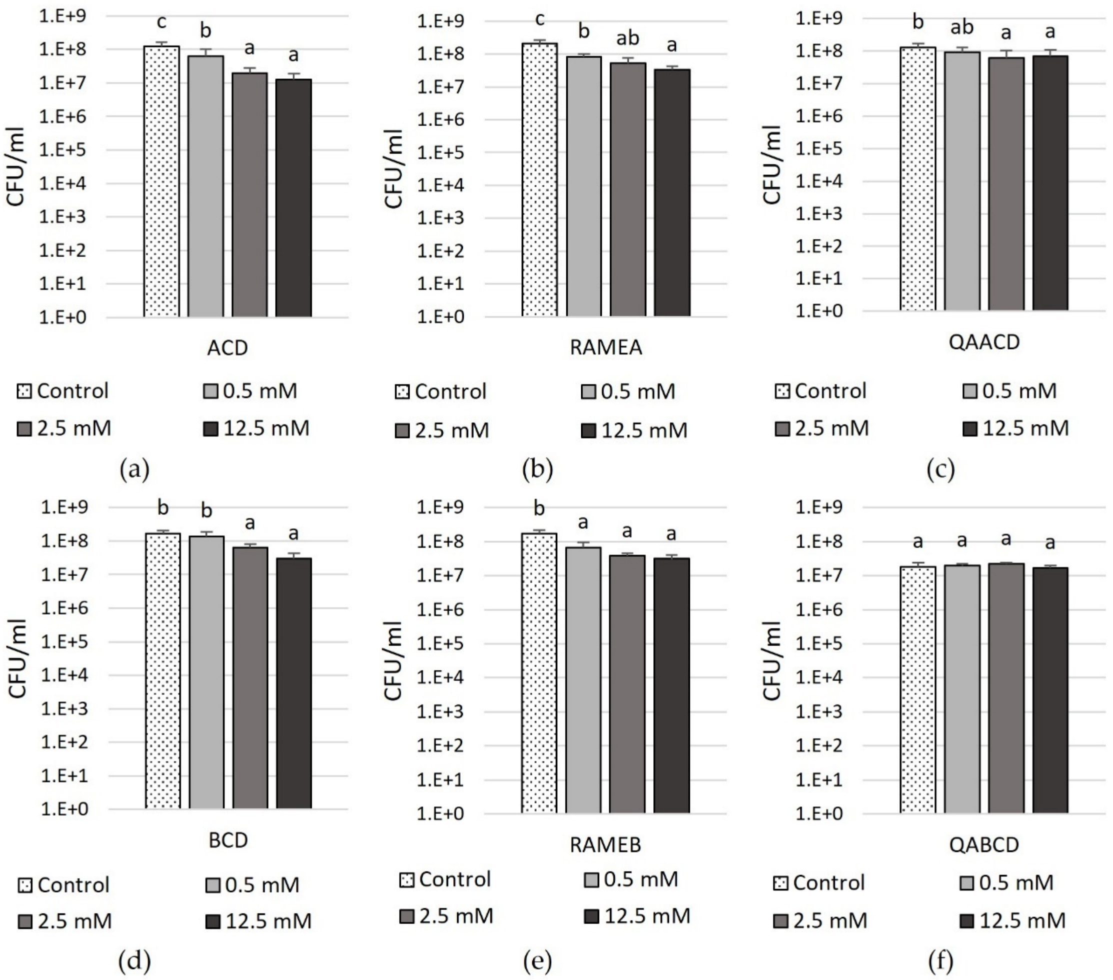

Supplementary Figure S1. The number of colonies formed by *Candida boidinii* (Colony Forming Units — CFU) after 24 hours of exposure time with different concentrations of CDs in the dynamic system: (a) ACD, (b) RAMEA, (c) QAACD, (d) BCD, (e) RAMEB, (f) QABCD. The data represents the averages of three replicates.

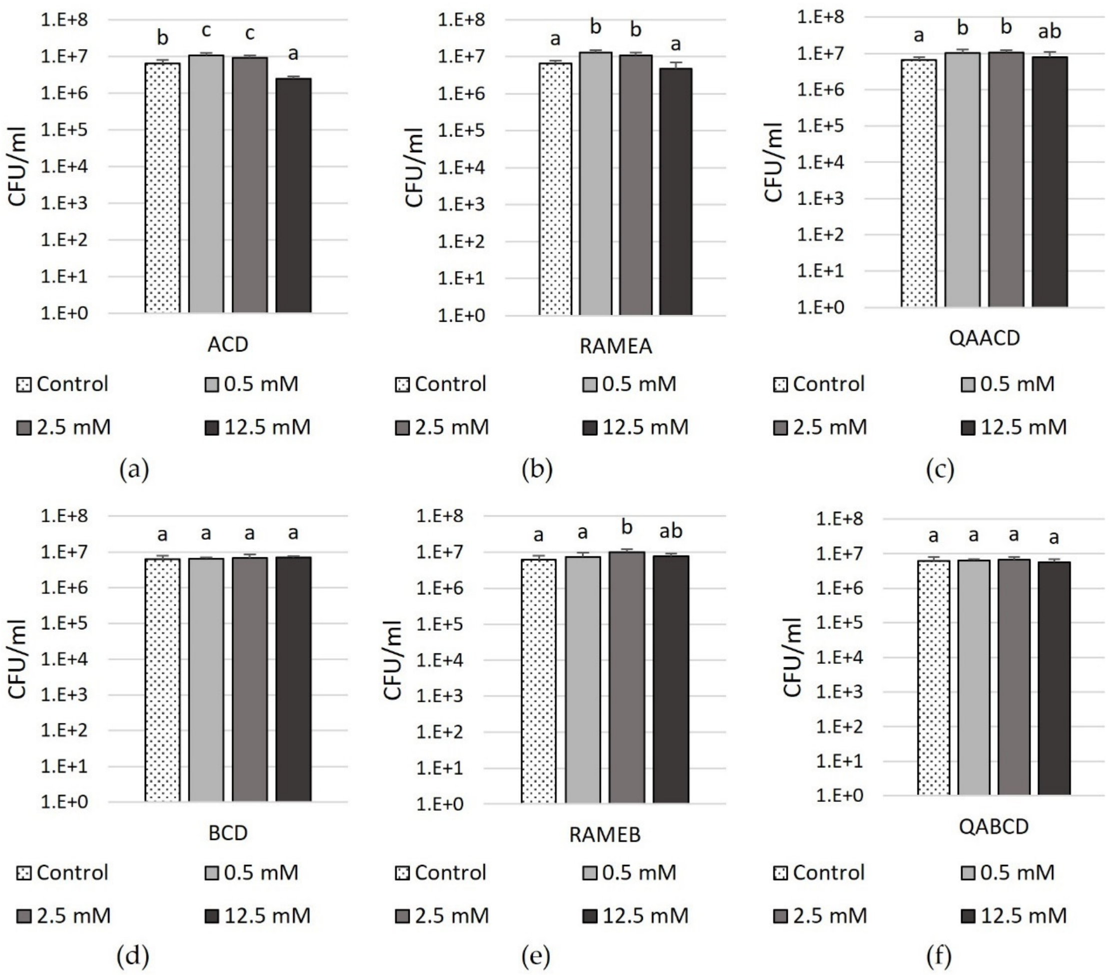

Supplementary Figure S2. The number of colonies formed by *Candida boidinii* (Colony Forming Units — CFU) after 24 hours of exposure time with different concentrations of CDs in the static system: (a) ACD, (b) RAMEA, (c) QAACD, (d) BCD, (e) RAMEB, (f) QABCD. The data represents the averages of three replicates.
